# Prevalence of analgesic refusal and reasons for refusal among children and parents in the pediatric emergency department

**DOI:** 10.1007/s00431-025-06465-2

**Published:** 2025-09-30

**Authors:** Giulia Secco, Maura Marin, Manuela Giangreco, Paola Devescovi, Nicoleta Stan, Susanna Zampa, Alessandro Amaddeo, Egidio Barbi, Giorgio Cozzi

**Affiliations:** 1 Department of Medicine, Surgery and Health Sciences, University of Trieste, Trieste, Italy; 2 Clinical Epidemiology and Public Health Research Unit, Institute for Maternal and Child Health, IRCCS “Burlo Garofolo”, Trieste, Italy; 3 Emergency Department, Institute for Maternal and Child Health—IRCCS Burlo Garofolo, Trieste, Italy

**Keywords:** Analgesic refusal, Pain management, Pain

## Abstract

Analgesic refusal in the emergency department can be a barrier to optimal pain management. This study aimed to quantify the prevalence of analgesic refusal among pediatric patients and their caregivers in this setting and to explore possible determinants and rationale for such refusal. This cross-sectional observational study was conducted at a tertiary-level pediatric emergency department in Italy from July to December 2023. A questionnaire was offered to children presenting with moderate to severe pain and their parents, collecting amnestic and clinical data. In case of analgesic refusal, the questionnaire asked for the reason. The primary outcome was the prevalence of analgesic refusal. Among 799 children with moderate to severe pain, 116 children refused the analgesia, yielding an overall refusal rate of 18.3%. Children with moderate pain, pain duration of 12–24 h, and those triaged as minor urgencies were significantly more likely to refuse analgesia. Conversely, children with severe pain, acute pain (< 12 h), and those triaged as urgent cases were more likely to accept analgesia. The main reason for refusing analgesia was that the perceived pain intensity was not severe enough to warrant pharmacological intervention. There were no significant differences in the characteristics of caregivers who accepted or refused analgesia.

*Conclusions*: This study found a high prevalence of pharmacological analgesia refusal in children presenting to the emergency department. Most refusals were due to the perception that the pain intensity was not severe enough to require pharmacological interventions.

**Table:** 

**What is Known:** • *Pain is a primary reason for Emergency Department (ED) visits in children.* • *Analgesic refusal can be seen as a barrier to optimal pain management in this setting.* • *No European studies have explored the prevalence of analgesic refusal in children and the reasons for refusal.* **What is New:** • *This study found an 18.3% prevalence of analgesic refusal among children with moderate to severe pain.* • *Children’s age, as well as the type and duration of pain, are variables associated with refusal.* • *Caregiver characteristics do not significantly influence the rate of refusal.* • *The primary reason for refusing analgesia was the perceived intensity of pain, supporting the notion that a simple numerical pain assessment may not accurately reflect a child's willingness to undergo pharmacological intervention.*

**What is Known:**

• *Pain is a primary reason for Emergency Department (ED) visits in children.*

• *Analgesic refusal can be seen as a barrier to optimal pain management in this setting.*

• *No European studies have explored the prevalence of analgesic refusal in children and the reasons for refusal.*

**What is New:**

• *This study found an 18.3% prevalence of analgesic refusal among children with moderate to severe pain.*

• *Children’s age, as well as the type and duration of pain, are variables associated with refusal.*

• *Caregiver characteristics do not significantly influence the rate of refusal.*

• *The primary reason for refusing analgesia was the perceived intensity of pain, supporting the notion that a simple numerical pain assessment may not accurately reflect a child's willingness to undergo pharmacological intervention.*

## Introduction

Pain is the most frequently complained symptom in children accessing the Pediatric Emergency Department (PED) [1], and its optimal management is one of the main goals for PED care.

Despite significant efforts over the past two decades to highlight the detrimental consequences of untreated pain in children worldwide [2–5], evidence shows that it remains underestimated and undertreated in some ED settings [5, 6].

Several barriers to the optimal management of children's pain in PEDs have been identified and addressed through staff education and protocol development [7].

In this context, a poorly explored barrier to pain treatment is related to caregiver or child refusal of analgesia offered by PED staff, with limited data available on this topic.

Two studies conducted in Canadian PEDs reported a prevalence of analgesic refusal of nearly 10%. They found that non-opioids were more frequently accepted as analgesics compared to opioids, mainly due to concerns about side effects and addiction [8, 9]. To our knowledge, no European data are available regarding the prevalence of analgesic refusal among children and caregivers in the PED.

Furthermore, no studies have directly investigated children's opinions or the reasons for their refusal.

This study aimed to evaluate the prevalence of analgesic refusal by children and caregivers in the PED of tertiary-level children's hospitals in Italy. Additionally, we sought to explore the main anamnestic and clinical features associated with analgesic refusal and to collect the key reported reasons for denial, with the goal of identifying and addressing potential barriers to optimal pain management in the PED.

## Methods

This cross-sectional observational clinical study was conducted at the tertiary-level university teaching children's hospital, the Institute for Maternal and Child Health IRCCS "Burlo Garofolo", Trieste, Italy, from July to December 2023.

The study protocol received ethical approval from the Institutional Review Board of the Institute (RC 37/24).

Eligible patients were children and adolescents, accompanied by a caregiver, who presented to the Pediatric Emergency Department (PED) of the Institute with moderate or severe pain.

Inclusion criteria were age between 0 and 17 years, moderate to severe pain at triage evaluation, (i.e., pain score of 4 to 10), and caregiver presence.

Exclusion criteria included patients requiring immediate medical evaluation, those with unstable vital signs or a reduced level of consciousness, patients with cognitive impairment, patients with a history suspected of physical or sexual abuse, patients with self-harm, and those whose caregivers were unable to understand the Italian language.

Study enrollment was carried out immediately after triage completion and any necessary offer and administration of analgesics. The decision to offer analgesics was made by the triage nurse and medical staff based on clinical need and was not influenced by participation in the study.

At triage, a specifically trained researcher presented and explained the study's aims to eligible patients and their caregivers. Patients were enrolled after obtaining written informed consent for participation in the study. To avoid temporal bias and ensure the selection of a representative sample, research personnel were available at various times of day, including night shifts, and on different days of the week, including weekends. Pain scores were evaluated, by the triage nurse, using validated rating scales based on the patients' age: the FLACC scale for infants and toddlers, the Wong-Baker scale for preschool children, and the numeric rating scale-11 (NRS-11) for children older than 7 years and older, including adolescents.

After triage, the researcher provided a questionnaire and was available to assist patients and caregivers with its completion if necessary. All questionnaires were administered by a single researcher. The questionnaire contained separate section for the caregiver and for patients aged 8 years or older.

The questionnaire was specifically designed for this study, as no pre-existing validated tool met the study’s objectives. It included 11 items covering caregivers’ demographic characteristics (age, gender, relationship to the child, nationality, and education level), as well as questions about the patient’s current episode of pain (source and duration of pain, use of medication at home, and acceptance or refusal of analgesics offered by PED staff). For the section related to analgesic refusal, two separate lists of refusal reasons were provided: one for caregivers and one for patients aged 8 years or older. Multiple refusal reasons could be selected.

Refusal was defined as the child not accepting the pharmacological analgesia offered at triage.

Demographic and clinical data were collected from the PED medical records for each patient.

All data were anonymized and collected in an electronic database developed specifically for this study.

For comparisons:

- Caregivers' age was grouped as follows: young adult (18–25 years), adult (26–44 years), middle-age (45–59 years), old age (60 years and over).
- Children's age was grouped as follows: infants (0–11 months), preschool children (1-5 years), school children (6–12 years), and adolescents (13–17 years).
- Type of pain was characterized as follows: injury-related pain (including pain from trauma, burns, and wounds), headache, otalgia, sore throat, abdominal pain, non-traumatic musculoskeletal pain, toothache, and others.
- Type of analgesic drugs included paracetamol, NSAIDs, opioids, ketamine, and others.

The primary study outcome was the prevalence of analgesic refusal among children in the PED. The secondary outcomes included the main reasons for analgesic refusal reported by children and caregivers, the prevalence of children offered analgesics by ED staff, and the demographic and clinical variables associated with analgesic refusal.

### Statistical analysis

Data from the current literature suggest that the prevalence of analgesic refusal by children in PEDs could be approximately 10% (Whiston CJEM 2018). Based on this estimate, the sample size required to estimate the prevalence of analgesic refusal by children in a PED, with a 95% confidence level and a 2.5% precision, was calculated to be 553 subjects.

Categorical variables were summarized using frequency and percentage. Continuous variables were summarized using the mean and standard deviation for normally distributed data, while the median and interquartile range (IQR) were used for non-normally distributed data. Normal distribution was assessed using the Kolmogorov–Smirnov test.

The prevalence of analgesic refusal was calculated as the ratio between the number of children who refuse analgesics and the total number of participants in the study.

The prevalence of children offered analgesics by ED staff during the ED visit was calculated as the ratio of children offered analgesics by ED staff to the total number of children.

The association between demographic or clinical variables and analgesic refusal was evaluated using the Chi-square test or Fisher’s exact test, as appropriate.

Univariate logistic regression model was performed with the analgesic refusal as dependent variable and the continuous pain score as independent variable. The ROC (Receiver Operating Characteristic) curve was constructed in the plane (1-specificity) * sensitivity, and the Youden’s index was calculated as the optimum cut-off. A p-value of less than 0.05 was considered statistically significant. All statistical analyses were performed using SAS 9.4 software (SAS Institute Inc., Cary, NC, USA).

## Results

During the study period, 2532 visits were recorded, 1689 of which involved children in pain. Among these, 799 (47.3%) presented with moderate to severe pain.

Pharmacological analgesia was offered to 638 children with moderate to severe pain, and 635 of them (79.5%) agreed to participate in the study. Tables 1 and 2 describe the main demographical and clinical features of children and caregivers enrolled in the study.

**Table 1:** Demographical and clinical features of children

	*n* = 635
***Gender*, *n* (%)**	
Male	366 (57.6%)
Female	269 (42.4%)
***Age*, *n* (%)**	
1–12 m	2 (0.3%)
1–5 y	93 (14.7%)
6–12 y	277 (43.6%)
13–17 y	263 (41.4%)
***Pain score*, *median (IQR)***	5 (4–7)
***Chronic disease*, *n* (%)**	23 (3.6%)
***Kind of pain*, *n* (%)**	
Injury-related pain	350 (55.1%)
Abdominal pain	58 (9.1%)
Otalgia	51 (8.0%)
Toothache	41 (6.5%)
Non-traumatic musculoskeletal pain	36 (5.7%)
Sore throat	27 (4.3%)
Headache	27 (4.3%)
Other	45 (7.1%)
***Triage code*, *n* (%)**	
No urgency	40 (6.3%)
Minor urgency	540 (85.0%)
Unavoidable urgency	53 (8.4%)
Emergency	2 (0.3%)
***Kind of analgesic offered in the ER*, *n* (%)**	
Ibuprofen	439 (69.1%)
Paracetamol	141 (22.2%)
Ketorolac	47 (7.4%)
Fentanyl	5 (0.8%)
Other	3 (0.5%)

**Table 2:** Demographical and clinical features of caregivers

	*n* = 635
***Gender*, *n* (%)**	
Male	211 (33.2%)
Female	424 (66.8%)
***Age*, *n* (%)**	
18–25 y	11 (1.7%)
26–44 y	297 (46.8%)
45–59 y	302 (47.6%)
> 60 y	25 (3.9%)
***Degree of kinship*, *n* (%)**	
Mother	394 (62.0%)
Father	196 (30.9%)
Other	45 (7.1%)
***Nationality*, *n* (%)**	
Italy	497 (78.3%)
Other countries	138 (21.7%)
***Degree*, *n* (%)**	
No educational degree	3 (0.5%)
Primary school	7 (1.1%)
Middle school	89 (14.0%)
High school	345 (54.3%)
Bachelor’s degree	184 (29.0%)
Doctoral degree	7 (1.1%)

Regarding the primary outcome of the study, 116 children refused the analgesia offered by the healthcare staff of the PED, resulting in an overall refusal rate of 18.3%.

Among them, 90 (77.6%) children had moderate pain, and 26 (22.4%) had severe pain.

Table 3 shows the comparisons between the children who accepted and refused the analgesia.

**Table 3:** Comparisons between child-caregiver pairs who accepted or refused the analgesia

	Analgesic accepted		
	NO (*n* = 116)	YES (*n* = 519)	*P* value
***Age,* n (%)**			** < *.0001***
1–12 m	0 (0.0%)	2 (0.4%)	1.00
1–5 y	7 (6.0%)	86 (16.6%)	0.0004
6–12 y	56 (48.3%)	221 (42.6%)	0.26
13–17 y	53 (45.7%)	210 (40.4%)	0.30
***Children gender*, *n* (%)**			***0.66***
Female	47 (40.5%)	222 (42.8%)	
Male	69 (59.5%)	297 (57.2%)	
***Chronic disease*, *n* (%)**			***0.15***
	1 (0.9%)	20 (3.9%)	
***Pain intensity*, *n* (%)**			***0.01***
Moderate	90 (77.6%)	339 (65.3%)	
Severe	26 (22.4%)	180 (34.7%)	
***Pain duration*, *n* (%)**			***0.01***
< 12 h	39 (33.9%)	250 (48.2%)	0.01
12–24 h	38 (33.0%)	105 (20.2%)	0.003
24–48 h	16 (13.9%)	46 (8.8%)	0.10
2–7 days	15 (13.0%)	88 (17.0%)	0.30
> 7 days	7 (6.1%)	30 (5.8%)	0.90
***Triage code*, *n* (%)**			** < *.0001***
No urgency	7 (6.0%)	33 (6.4%)	0.90
Minor urgency	108 (93.1%)	432 (83.2%)	0.07
Unavoidable urgency	1 (0.9%)	52 (10.0%)	0.001
Emergency	0 (0.0%)	2 (0.4%)	1.00
***Kind of analgesic offered in the ER*, *n* (%)**			** < *.0001***
Ibuprofen	93 (80.2%)	346 (66.7%)	0.004
Acetaminophen	23 (19.8%)	118 (22.7%)	0.50
Ketorolac	0 (0.0%)	47 (9.1%)	< 0.0001
Fentanyl	0 (0.0%)	5 (1.0%)	0.59
Other	0 (0.0%)	3 (0.6%)	1.00
***Kind of pain*, *n* (%)**			**0.01**
Injury-related pain	86 (74.1)	264 (50.9)	<.0001
Abdominal pain	9 (7.8)	49 (9.4)	0.57
Otalgia	2 (1.7)	49 (9.4)	0.004
Toothache	4 (3.4)	37 (7.1)	0.21
Non-traumatic musculoskeletal pain	6 (5.2)	30 (5.8)	0.80
Sore throat	2 (1.7)	25 (4.8)	0.20
Headache	2 (1.7)	25 (4.8)	0.20
Other	5 (4.3)	40 (7.7)	0.20
***Age caregivers*, *n* (%)**			**0.61**
18–25	1 (0.9)	10 (1.9)	0.70
26–44	50 (43.5)	246 (47.4)	0.45
45–59	61 (53.0)	241 (46.4)	0.20
> = 60	3 (2.6)	22 (4.2)	0.60
***Gender caregivers*, *n* (%)**			**0.17**
Male	32 (27.8)	179 (34.5)	
Female	83 (72.2)	340 (65.5)	
***Nationality caregivers*, *n* (%)**			**0.31**
Italy	94 (81.7)	402 (77.5)	
Other countries	21 (18.3)	117 (22.5)	
***Degree caregivers*, *n* (%)**			**0.42**
No educational degree	0 (0.0)	3 (0.6)	1.00
Primary school	2 (1.7)	5 (1.0)	0.62
Middle school	11 (9.6)	78 (15.0)	0.13
High school	68 (59.1)	276 (53.2)	0.25
Bachelor’s degree	32 (27.8)	152 (29.3)	0.76
Doctoral degree	2 (1.7)	5 (1.0)	0.62

Preschool children were more likely to take the analgesic. No differences were found among the demographical characteristics of caregivers whose children accepted or refused analgesia.

Children with moderate pain, pain lasting 12- 24 h, injury-related pain, or triaged as minor urgencies, were significantly more likely to refuse analgesia. In contrast, children with severe pain, more acute pain (< 12 h), otalgia, or triaged as urgencies were significantly more likely to accept analgesia.

The probability of refusal decreased by 16% for each one-point increase in pain score (OR = 0.84; 95% CI: 0.73–0.96), with an optimal pain score cut-off of 6 according to the Youden index.

Table 4 presents the main reasons for refusal reported by children and caregivers.

**Table 4:** The main reasons for refusal declared by children and caregivers

*Caregiver refusal motivation*	*n* = 116
I don’t think my child is experiencing such severe pain	47 (40.5%)
I believe my child needs to undergo some medical examinations before proceeding with any treatment	12 (10.3%)
I let my child decide	17 (14.7%)
My child doesn’t like taking medicine	11 (9.5%)
Generally, I do not give my child pain medication for this condition	8 (6.9%)
My child has a high pain tolerance	4 (3.5%)
I think it’s harder for the doctor to make a diagnosis with pain medication	6 (5.2%)
I'm concerned about the side effects of pain medication	1 (0.9%)
I already gave my child pain medication at home	3 (2.6%)
The doctor should decide whether to give the pain medication	2 (1.7%)
Other motivation	5 (4.3%)
***Child refusal motivation (*> *8 y)***	***n* = 98**
I don’t feel that much pain	58 (59.2%)
I don’t like taking medications	10 (10.2%)
I have a high pain tolerance	4 (4.1%)
I usually don’t take analgesics for this condition	6 (6.1%)
I can’t swallow the pill/the pill is too big	2 (2.0%)
I don’t think analgesics are very helpful	1 (1.0%)
The medication makes me nauseous	1 (1.0%)
I’m worried about the potential side effects of analgesics	2 (2.0%)
No one explained to me what the medication is for	1 (1.0%)
Other motivation	13 (13.3%)

The most frequently reported reason for refusal, both for children > 8 years old and caregivers, was that the intensity of pain was not severe enough to warrant pharmacological analgesia.

Among the 26 children with severe pain who refused the analgesic, 14 (54%) justified their decision with statements indicating that their pain was not so severe to require pharmacological treatment.

Only 2% of children and 0.9% of caregivers refused due to concerns about possible adverse effects of the drugs.

No differences were found in the provision of analgesia by ED staff, except for preschool children, for whom analgesia was significantly less frequently offered. Similarly, children with moderate pain were less likely to be offered analgesia (Supplementary Material: Table 1s).

## Discussion

This study showed a prevalence of analgesic refusal at the triage of our children's hospital PED of 18%. This prevalence was considerably higher than the nearly 10% reported in a previous Canadian study (Whiston). It should be noted that the two studies are heterogeneous in many respects, which may have contributed to this discrepancy. In this study, we considered all causes and durations of pain, while Whilston et al. considered only specific pain causes and patients with pain lasting less than 24 h. Moreover, the median pain score in our series was five, compared to eight in the Canadian study. Finally, we considered only pharmacological analgesia and did not record the use of non-pharmacological techniques for pain management.

Since appropriate pain assessment and management are primary goals of pediatric care, investigating the limitations to achieving this is crucial. Although our study findings suggest that analgesic refusal could represent a common cause of inadequate analgesia, we should remember that evidence from studies on adult patients has shown that patient-reported pain scores do not accurately predict the desire for pharmacological analgesia [10–12].

Singer et al. reported that nearly half of ED patients with moderate to severe pain did not desire analgesics [10]. Similarly, Allione et al. found that 48.6% of their ED patients refused pharmacological analgesia [13].

Evidence in the pediatric population regarding this topic remains limited. The majority of studies investigating the association between pain scores and the need for pharmacological analgesia have not been conducted in emergency department (ED) settings and have predominantly involved children experiencing postoperative pain [14–16].

A single study conducted in the ED sought to determine a pain score threshold that accurately reflects a child's perceived need for pharmacological analgesia. The authors reported a median pain score of 6 on a 0–10 numeric scale as indicative of analgesic requirement. However, in that study, children were asked to hypothetically indicate the level of pain at which they would have required analgesia, irrespective of their actual pain intensity or clinical condition at the time of the interview [17]. Despite these methodological limitations, our findings are consistent with the results of this previous investigation.

In recent decades, significant efforts have been made to educate PED staff and caregivers on the importance of accurate pain measurement and adequate pain management worldwide [18].

Notable improvements have been achieved by highlighting the detrimental consequences of oligo analgesia in children. Several studies have shown that adding mandatory mandated pain scoring during ED triage resulted in more frequent analgesic administration [19, 20].

Nevertheless, clinicians should remember that pain scoring is only part of the pain evaluation process—a distinct clinical task that includes history, physical examination, and appropriate diagnostic evaluation (including the consideration of malingering or somatic pain in older children).

As reported by Voepel-Lewis, an individualized approach to interpreting pain scores in children should take into account the clinical context and the medical source of pain—both of which may influence the child’s self-report—as well as the assessment of physiological and behavioral indicators, and the child's potential needs and goals [21]. In this context, astute clinicians should be aware that pain scores may not accurately identify patients who want analgesia, and a numerical measure of pain at triage should not be considered equivalent to comprehensive pain assessment [12]. A Patient's anamnestic and physiological features should be considered when choosing the appropriate pharmacological analgesia to administer immediately.

One of the main strengths of this study is that it is the first to directly investigate the reasons for analgesic refusal among children. The results indicated that the primary reason for refusal was related to the intensity of the pain itself, confirming that a simple numerical measure of pain should not be used as the sole tool to assess the need for or desire for pharmacological analgesia in children, as already reported among adults.

Furthermore, we found that refusals were associated with specific causes of pain, particularly injury-related pain. These data support the knowledge that certain pain causes may benefit more from non-pharmacological techniques than others.

As with children, the main reason for refusal by caregivers was that the pain intensity reported by the children was not high enough to require pharmacological analgesia. Naturally, we cannot exclude the potential influence of caregivers on their children’s judgement regarding pain intensity and the need for pharmacological analgesia. However, we did not identify any specific profile for caregivers whose children refused analgesia. In particular, there were no differences in gender, age, geographical origin, or education level between caregivers of children who accepted or refused analgesia. Therefore, it seems that no specific caregiver characteristics influence the refusal rate. Remarkably, a considerable proportion (5.2%) of caregivers still believe that pharmacological analgesia can interfere with the diagnostic process.

From this perspective, further staff training is needed to improve communication with children and caregivers during triage and promote the acceptance of pharmacological analgesia.

Overall, these data suggest that the rate of pharmacological analgesia provided should not be considered an absolute measure of appropriate pain management in the PED setting.

This study has some limitations. First, being a single-center experience, local factors may have influenced the results, and the findings should be cautiously generalized to other settings.

Additionally, patient enrolment was not consecutive but depended on the availability of research personnel, so we cannot exclude some selection bias. We did not offer a questionnaire investigating the reasons for refusal to children younger than 8 years old, as we wanted to ensure they were capable of reading and completing the questionnaire independently. Finally, only a few infants were enrolled, making it difficult to draw conclusions for this patient age group.

In conclusion, this study found a considerable prevalence of pharmacological analgesia refusal among children in the PED of a tertiary-level children’s hospital. Children with moderate pain, injury-related pain, and pain lasting longer than 24 h were more likely to refuse analgesia. The main reason for refusal was the perception that the pain was not severe enough to require pharmacological analgesia. Further multicenter studies are needed to confirm these findings.

## Data Availability

No datasets were generated or analysed during the current study.
